# Supplementary material for: Histidine-Rich Glycoprotein Suppresses the S100A8/A9-Mediated Organotropic Metastasis of Melanoma Cells
Source: Int J Mol Sci. 2022 Sep 7;23(18):10300. doi: 10.3390/ijms231810300 (PMC9499646; doi:10.3390/ijms231810300)
Supplement: Supplementary file 1 [file ijms-23-10300-s001.zip › ijms-1884637-supplementary.pdf]

Supplementary Information

Tomonobu et al.

Supplementary Figure S1

BP1

Group (#)	Spectra (#)	Distinct Peptides (#)	Distinct Summed MS/MS Search Score	% AA Coverage	Mean Peptide Spectral Intensity	Protein MW (Da)	Protein pI	Species	Database Accession #	Protein Name
1	134	15	277.01	32.1	4.77e+008	60563.9	7.09	HUMAN	P04196	Histidine-rich glycoprotein
2	1	1	13.02	0.9	9.03e+007	155869.0	5.52	HUMAN	Q99683	Mitogen-activated protein kinase kinase kinase 5
Totals:	135	16								

HRG

BP2

Group (#)	Spectra (#)	Distinct Peptides (#)	Distinct Summed MS/MS Search Score	% AA Coverage	Mean Peptide Spectral Intensity	Protein MW (Da)	Protein pI	Species	Database Accession #	Protein Name
1	139	16	281.84	32.3	5.39e+008	60563.9	7.09	HUMAN	P04196	Histidine-rich glycoprotein
Totals:	139	16								

HRG

BP3

Group (#)	Spectra (#)	Distinct Peptides (#)	Distinct Summed MS/MS Search Score	% AA Coverage	Mean Peptide Spectral Intensity	Protein MW (Da)	Protein pI	Species	Database Accession #	Protein Name
1	124	14	251.87	28.3	5.68e+008	60563.9	7.09	HUMAN	P04196	Histidine-rich glycoprotein
2	1	1	13.83	0.9	9.81e+007	155869.0	5.52	HUMAN	Q99683	Mitogen-activated protein kinase kinase kinase 5
3	1	1	13.59	3	9.78e+008	79990.4	7.85	HUMAN	P29973	cGMP-gated cation channel alpha-1
Totals:	126	16								

HRG

Supplementary figure legends

Figure S1. Identification of protein(s) interacting with S100A8/A9. Three bands, BP1, 2 and 3, clearly stained with CBB, that bound with the single GST-S100A9 as well as the mixture of GST-S100A8 and GST-S100A9 in a consistent manner, were subjected to a liquid chromatography–tandem mass spectrometry (LC-MS/MS) analysis; the results are displayed as a modified list.

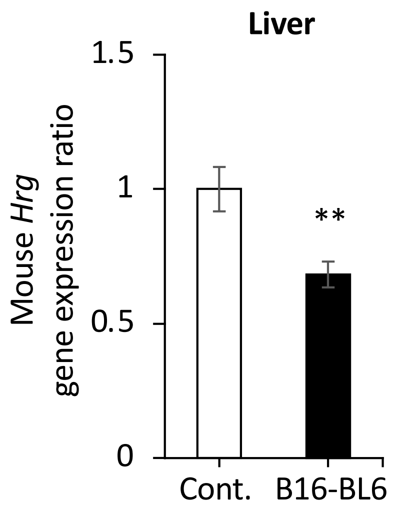

Figure S2. Change in the expression of HRG in melanoma-burdened mice. Because the liver is the main source of HRG production, the livers were also resected from mouse melanoma (B16-BL6 cells)-burdened C57BL/6J mice at the same time point (Day 7) as in the experiment shown in Figure 4A. The resected livers were analyzed for *Hrg* gene expression levels. *Tbp* mRNA was used as a control for the analysis. The abbreviation Cont. represents healthy liver from the non-tumor burdened control mice. B16-BL6 represents a diseased liver from mouse melanoma (B16-BL6 cells)-burdened mice.
